# The major biogenic amine metabolites in mood disorders

**DOI:** 10.3389/fpsyt.2024.1460631

**Published:** 2024-09-24

**Authors:** Jingyi Yang, Minlan Yuan, Wei Zhang

**Affiliations:** ^1^ Mental Health Center and Psychiatric Laboratory, the State Key Laboratory of Biotherapy, West China Hospital, Sichuan University, Chengdu, China; ^2^ West China Biomedical Big Data Center, West China Hospital, Sichuan University, Chengdu, China; ^3^ Big Data Center, Sichuan University, Chengdu, China

**Keywords:** mood disorder, major depressive disorder, bipolar disorder, metabolite, biogenic amine

## Abstract

Mood disorders, including major depressive disorder and bipolar disorder, have a profound impact on more than 300 million people worldwide. It has been demonstrated mood disorders were closely associated with deviations in biogenic amine metabolites, which are involved in numerous critical physiological processes. The peripheral and central alteration of biogenic amine metabolites in patients may be one of the potential pathogeneses of mood disorders. This review provides a concise overview of the latest research on biogenic amine metabolites in mood disorders, such as histamine, kynurenine, and creatine. Further studies need larger sample sizes and multi-center collaboration. Investigating the changes of biogenic amine metabolites in mood disorders can provide biological foundation for diagnosis, offer guidance for more potent treatments, and aid in elucidating the biological mechanisms underlying mood disorders.

## 1 Introduction

Mood disorder is a kind of chronic mental disorder, which has a great impact on people’s physical and mental health. The broadly defined mood disorders include major depressive disorder, bipolar disorder, dysthymia and other diseases (1, 2), while major depressive disorder and bipolar disorder, known as narrowly defined mood disorders, impact approximately 20% of population worldwide, placing a huge burden on the patients and society (3–6). Mood disorders, characterized by significant fluctuations in mood and sleep disturbances, can lead to suicidal tendencies. It is estimated that up to 50% of the 800,000 suicides worldwide each year occur during periods of depressive episodes (7, 8). The possible pathophysiology of major depressive illness has been attributed to neuroinflammation, hormones, and hereditary factors (9, 10). On the other hand, bipolar disorder seems to be caused by internal clock anomalies driven by low-grade inflammation (11). Studying the pathophysiology of mood disorders not only leads to more promising treatments, but also offers proof that the illnesses can be prevented altogether.

Prior research has demonstrated an extensive connection between mental health and physical fitness, with the biochemical alterations in the peripheral system potentially contributing to the development of mood disorders (12–14). The metabolites were reported to supply the substrates for biological processes and transmit vital signals via regulating other regulatory proteins (15), indicating whether or not the body’s physiological functions are proper. Biogenic amines, including catecholamines, indole amines and histamines, are nitrogen-containing compounds produced by α-decarboxylation of amino acids (16). Biogenic amines are highly prevalent in human tissues and are crucial for maintaining DNA replication, hormone synthesis, protein synthesis, cell activity, and other regular physiological processes (17). In the human body, biogenic amine metabolites, such as taurine, aniline, betaine, choline, histamine, kynurenine, and creatine, can respond to changes in cellular metabolism and provide insights into the normalcy of the physiological processes (18). In recent years, it has been discovered that these metabolites have an intimate connection with mental illnesses, particularly mood disorders (14, 19). Exploring the alterations in biogenic amine metabolites in the peripheral and central nervous systems of individuals suffering from mood disorders may not only offer an innovative approach to identify the conditions, but also shed light on the biological mechanisms underlying mood disorders and pave the way for the discovery of novel therapeutic targets in the future.

## 2 Histamine and mood disorders

Histamine, a metabolite of histidine, is expelled from mast cells in response to tissue damage or allergic reactions (20). As a transmitter in the nervous system and a signaling molecule in the gut, skin and immune system (21), histamine mainly exists in neuroepithelial cells and hematopoietic cells, and plays a great role in gastric acid secretion, immune regulation, bronchus smooth muscle contraction, and blood vessel dilation (21). The axons of histaminergic neurons predominantly reside in the posterior tuberomammillary nucleus of the hypothalamus (22). Histamine binds to the brain primarily through G-protein-coupled receptors, of which the brain contains four types: H1, H2, H3, and H4. (**Table 1**).

**Table 1:** The distribution and function of histamine receptors in human body.

Receptor	Distribution	Effects
**H1 Receptor**	Central nervous system, especially in thalamus, cortex and other regions (210)	Activate neurons and astrocytes (211); Activate H1R, leading to the production of cyclic guanosine monophosphate (cGMP) and nitric oxide (NO), thus inducing the production of arachidonic acid (21, 23)
**H2 Receptor**	Mainly in basal ganglia, hippocampus, amygdala and cerebral cortex (210, 212)	Induce an increase in intracellular cyclic adenosine phosphate (cAMP) production (41); Block Ca^2+^ activated potassium conduction and inhibit the release of PLA2 and arachidonic acid (21, 23)
**H3 Receptor**	Widely in the central nervous system, especially in the cortex, hippocampus and caudate nucleus, but low in peripheral tissues (213, 214)	Mediate feedback inhibition of histamine release and synthesis, and regulate the release of other neurotransmitters such as dopamine and GABA (215)
**H4 Receptor**	Mainly on hematopoietic cells and immune cells such as mast cells, eosinophils, dendritic cells, and microglia (41)	Play a role in histamine-mediated inflammation, but the specific mechanism is still unclear (41)

### 2.1 Major depressive disorder

As a neurotransmitter, histamine regulates several important brain functions, including cognition and mood (23). A clinical study showed that adolescents diagnosed with major depressive disorder exhibited notably elevated levels of peripheral histamine compared to healthy individuals (24). Not only peripheral histamine levels are different, but also histamine receptor levels in the brain of patients with depressive disorder are differentially expressed. The degree of the reduction in H1 receptor binding was found to be linked with the intensity of depression symptoms in the prefrontal cortex, frontal cortex, and cingulate gyrus (25), indicating that H1 receptors could be potential targets for treatment of major depressive disorder (26).

Histamine receptor inhibitors and inverse agonists have been demonstrated in numerous animal experiments to have antidepressant effects in mice (27–31) (**Table 2**). Previous studies found that adult mice lacking brain histamine exhibited indications of depression and reduced levels of activity (32). Selective serotonin reuptake inhibitors (SSRIs) such as citalopram have limited efficacy in treating depressive symptoms in mice with histamine synthesis disorders (33), suggesting that SSRIs antidepressants require a complete histamine system to be clinically effective. In addition, compared to normal mice, the mast cell-free mice reported elevated levels of anxiety and despair (34). Experiments revealed that inhibiting the activity of the H3 receptor using H3 receptor antagonists resulted in elevated levels of histamine, which in turn led to an upregulation of BDNF expression in the prefrontal and hippocampal regions of depressed mice via the H4 receptor. Additionally, this intervention normalized the overactive HPA axis, demonstrating an antidepressant effect (30).

**Table 2:** Studies of histamine receptors in depression animal models.

Author	Target Receptor	Main conclusion
**Iida, et al (** 27)	H3 Receptor	The H3 receptor inverse agonist JNJ reduced LPS-induced upregulation of microglia proinflammatory cytokines and improved depression-like behavior.
**Bah, et al.** (28)	H3 Receptor	The H3 receptor antagonist ST-1283 can improve anxiety and depressive behavior in mice, and the pretreatment of the H3 receptor agonist R-alpha-methylhistamine can eliminate the antianxiety and antidepressant effects of ST-1283.
**Femenía, et al.** (29)	H1、H2、H3 Receptor	The H3 receptor antagonist chlorfenpropyl alleviated depression, reversed memory deficits, and increased hippocampal GluN2A protein levels in rats. The antidepressant effects of clopropyl and the enhancement of synaptic plasticity require activation of H1 and H2 receptors in the hippocampus.
**Kumar, et al.** (30)	H3、H4 Receptor	The H3 receptor antagonist ciproxifan alleviated frontal depression-like symptoms induced by chronic unpredictable stress and increased the expression of BDNF in the prefrontal cortex (PFC) and hippocampus of depressed mice. Histamine can significantly induce BDNF expression, and H4 receptor selective antagonists block BDNF expression, while other histamine receptor selective antagonists do not block BDNF expression.
**Sanna, et al.** (31)	H4 Receptor	The H4 receptor knockout mice showed significant symptoms of anxiety and depression, and the immobile time increased in the tail suspension experiment.

However, early animal studies have shown that acute and chronic restraint stress elevate histamine turnover in the nucleus accumbens and striatum in rats (35, 36). After forced swimming experiment, there was a notable rise in the concentration of the histamine metabolite tele-methylhistamine in the cerebral cortex of mice. Antagonizing the H1 receptor may have an antidepressant effect since the rats’ forced swimming experiment’s rest period decreased after the H1 receptor antagonist was administered (37). In addition, the H3 receptor antagonist thioperamide can trigger the release of histamine in neurons, and the increased histamine stimulates the H1 receptor to mediate the anxiety effect, causing the mice exhibiting overt anxiety symptoms in the light-dark box experiment (38). H1 receptor antagonists injected into the lateral interventricular septum (39) and basolateral amygdala (40) significantly reversed anxiety-like behavior in rats. The aforementioned research demonstrates that both acute and long-term stress raise histamine turnover in the brain, which may be connected to the etiology of anxiety. It also reveals that anxiety symptoms may be mediated by the activation of the H1 receptor.

### 2.2 Bipolar disorder

The histaminergic system is involved in many basic physiological functions, including the sleep-wake cycle, which is strongly associated with bipolar disorder (41). Sectional analysis of the brains of bipolar disorder patients found that histamine H3 receptors in the prefrontal cortex may be involved in the regulation of cognitive impairment by regulating the connections between the hippocampus and various cortical and subcortical regions (22).

## 3 Kynurenine pathway and mood disorders

**Figure 1:**
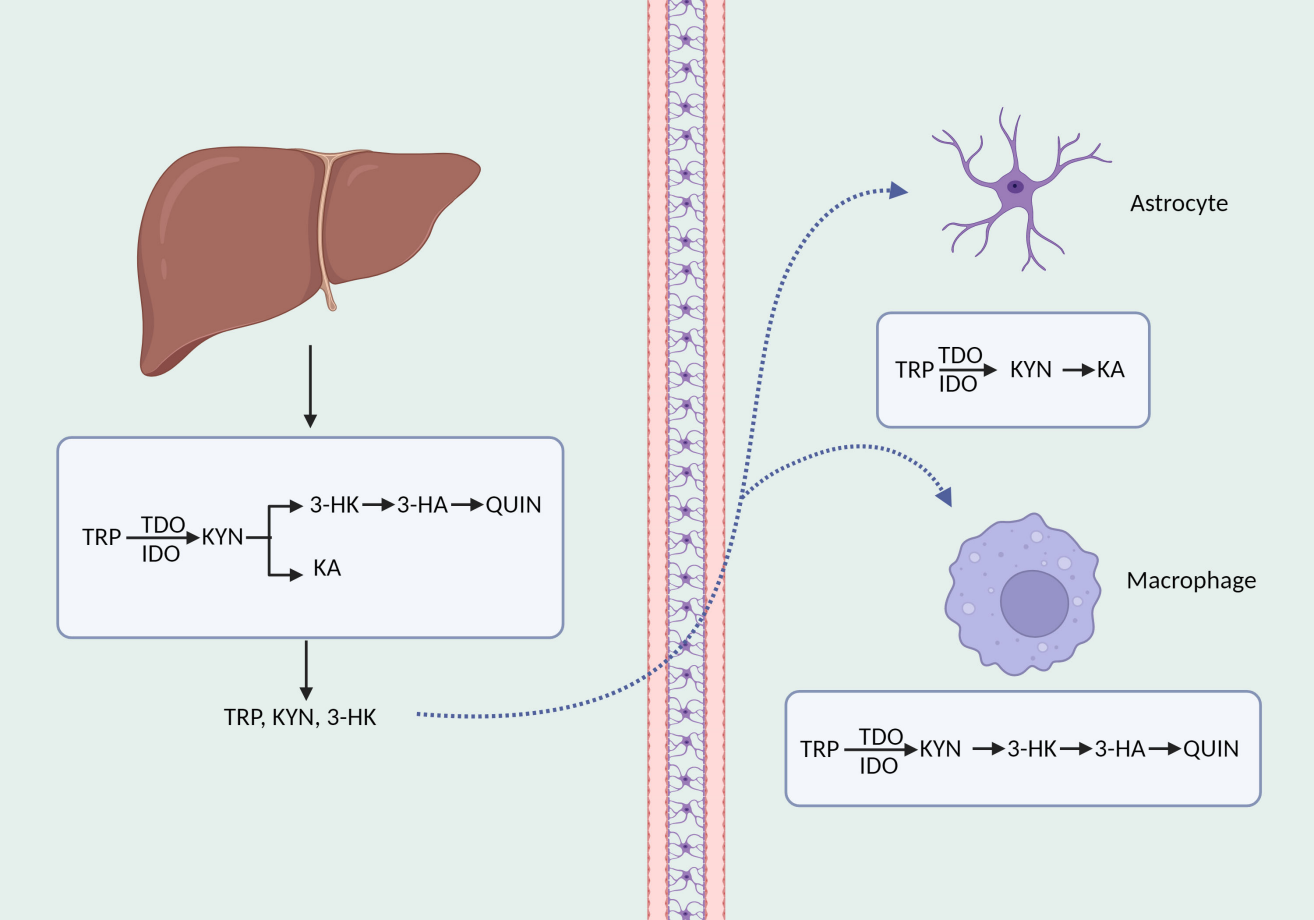
The synthesis and decomposition of kynurenine. TRP, tryptophan; KYN, kynurenine; QUIN, quinolinic acid; KA, kynurenic acid.

Tryptophan, an essential amino acid, forms a vary of neuroactive compounds with physiologically significant roles. Apart from the process of serotonin production, the kynurenine pathway is responsible for the metabolism of approximately 90% (42) of tryptophan. The kynurenine route commences with the transformation of tryptophan into kynurenine, which is the initial and rate-limiting step, facilitated by indoleamine 2, 3-dioxygenase (IDO) 1, IDO2, and tryptophan 2, 3-dioxygenase (TDO) (43). Numerous immune system cells express the IDO1 enzyme, such as macrophages, astrocytes, and microglia (44). Compared to IDO1 enzyme, IDO2 enzyme is mainly located in the liver and kidney with a reduced catalytic activity (45). Additionally, TDO is mostly found in the liver and can be preferentially triggered by glucocorticoids as well as low concentrations of reactive oxygen species (46). 60% of kynurenic acid in the central nervous system originates from the periphery and is able to cross the blood-brain barrier by utilizing neutral amino acid transporters (47). Kynurenine aminotransferase (KAT) I, II, III, and IV catalyzes the conversion of kynurenine in astrocytes to kynurenic acid, which is believed to have a neuroprotective impact against the excitotoxic and apoptotic effects of NMDA receptors (48, 49). In microglia and macrophages, kynurenine is progressively degraded to 3-hydroxykynurenine (3-HK), 3-hydroxycyanuric acid (3-HA), and ultimately converted to neurotoxic quinolinic acid (48)(**Figure 1**).

### 3.1 Major depressive disorder

Kynurenine pathway metabolites are intimately related to inflammatory pathways, and recent studies indicated that disruption of the kynurenine pathway could potentially contribute to the etiology of depression (50, 51). Numerous studies have examined the concentrations of metabolites in the peripheral kynurenine pathway in patients diagnosed with depression, and found that in comparison to healthy controls, patients with depression had lower levels of peripheral tryptophan, kynurenine, and kynurenine quinolinic acid (52–55), a lower ratio of kynurenine to kynurenine (56), and a higher ratio of kynurenine to tryptophan (57, 58). These findings suggest that major depressive disorder could be caused by an imbalance between neuroprotective and neurotoxic metabolites in the kynurenine pathway (54, 59). Previous study found that individuals with endogenous anxiety had higher plasma kynurenine concentrations than patients with endogenous depression, which suggests that plasma kynurenine can be used as an additional standard to discriminate between the two conditions (60). The severity of depression in individuals with postpartum depression was found to be positively correlated with elevated levels of plasma IL-6 and IL-8, as well as reduced levels of quinolinic acid (61).

Since kynurenine pathway metabolites can penetrate the blood-brain barrier, alterations in metabolite levels can affect central neurotransmitter signaling. A favorable association was seen between the concentration of plasma kynurenine, the 3-HAA content in the right caudate nucleus, and the total choline in the left putamina striatum in an adolescent depressive disorder research (62). In patients with depressive disorder, the ratio of serum kynurenic acid to 3-hydroxykynurenine was reported to be inversely linked with left hippocampus activity during autobiographical memory recall (63). Studies have shown that the ratio of serum kynurequinolinic acid to quinolinic acid in patients with depressive disorder is negatively connected with the severity of depressive symptoms (64, 65), but positively correlated with hippocampus volume (66). Following the administration of interferon-gamma, the concentrations of kynurenine, kynurenquinolinic acid, and quinolinic acid in the cerebral fluid of patients with hepatitis C were elevated, and were substantially linked to depressed symptoms (50).

At present, studies on the kynurenine pathway metabolites in the brain tissue of patients with depressive disorders are very limited. Patients with depressive disorders exhibited a notable reduction in the activation of the kynurenine pathway, as well as decreased levels of quinolinic acid and expression of the IDO1 and IDO2 enzymes in the ventral prefrontal cortex (67). Furthermore, studies have demonstrated that patients with major depressive disorder had higher densities of quinoline-positive microglia in both the anterior and premedia cingulate cortex (68), which further suggests certain regional specificity (69).

### 3.2 Bipolar disorder

In recent years, the equilibrium of the kynurenine pathway’s neuroprotective and neurotoxic effects has been proposed as one of the potential physiological underpinnings of bipolar disorder. Compared with healthy people, the levels of peripheral tryptophan, kynurenine and kynurenquinolinic acid decreased in BD patients, and the levels of tryptophan decreased the most in individuals with manic episodes (70). Peripheral 3-HK levels were associated with the severity of depressive episodes in patients with bipolar disorder (71), while peripheral kynurequinolinic acid concentrations had a weakly negative correlation with depressed episodes (72). A study conducted on adolescent patients revealed a favorable association between depression symptoms and the kynurenic acid/kynurenine ratio, as well as a negative correlation between depression symptoms with the kynurenine and kynurenine/tryptophan ratio (73). Peripheral kynurenine and kynurenic acid levels rose dramatically following ketamine administration, whereas the quinolinic acid to kynurenine ratio fell (74).

Studies have indicated that in bipolar disorder patients, there is a correlation between the integrity of white matter in multiple brain regions, including the corpus callosum, and the decreased level of peripheral kynurenic acid (75), the increased level of kynurenic acid, and the increased kynurenic acid/tryptophan ratio (76). Decreased levels of tryptophan are associated with depressive symptoms, but increased amounts of kynurenic acid may safeguard the structure of white matter in the brain. Activation of the kynurenine pathway may be cohort-specific in bipolar disorder patients, though, as evidenced by the lack of a statistically significant correlation between various metabolites of the kynurenine pathway in cerebrospinal fluid and depressive symptoms in bipolar disorder patients (77)(**Table 3**).

**Table 3:** Studies of kynurenine pathway metabolites in mood disorders.

	Location	Main finding
**Major Depressive Disorder**	Peripheral system	Tryptophan↓, Kynurenine↓, Kynurenic acid↓ (53–55), Kynurenic acid/Kynurenine↓ (56), Kynurenine/Tryptophan↑ (57, 58)
	Central system	Ventral prefrontal cortex kynurenic acid↓ (67) Anterior cingulate cortex, premedial cingulate cortex kynurenic acid↑ (68)
**Bipolar Disorder**	Peripheral system	Tryptophan↓, Kynurenine↓, Kynurenic acid↓ (71, 75), Kynurenine/Tryptophan↓ (73) 3-hydroxykynurenine↑ (71), Kynurenic acid/Kynurenine↑ (73)

↑ means the level of the metabolite was higher than healthy controls. ↓ means that the level of the metabolite was lower than healthy controls.

## 4 Creatine/creatine kinase pathway and mood disorders

**Figure 2:**
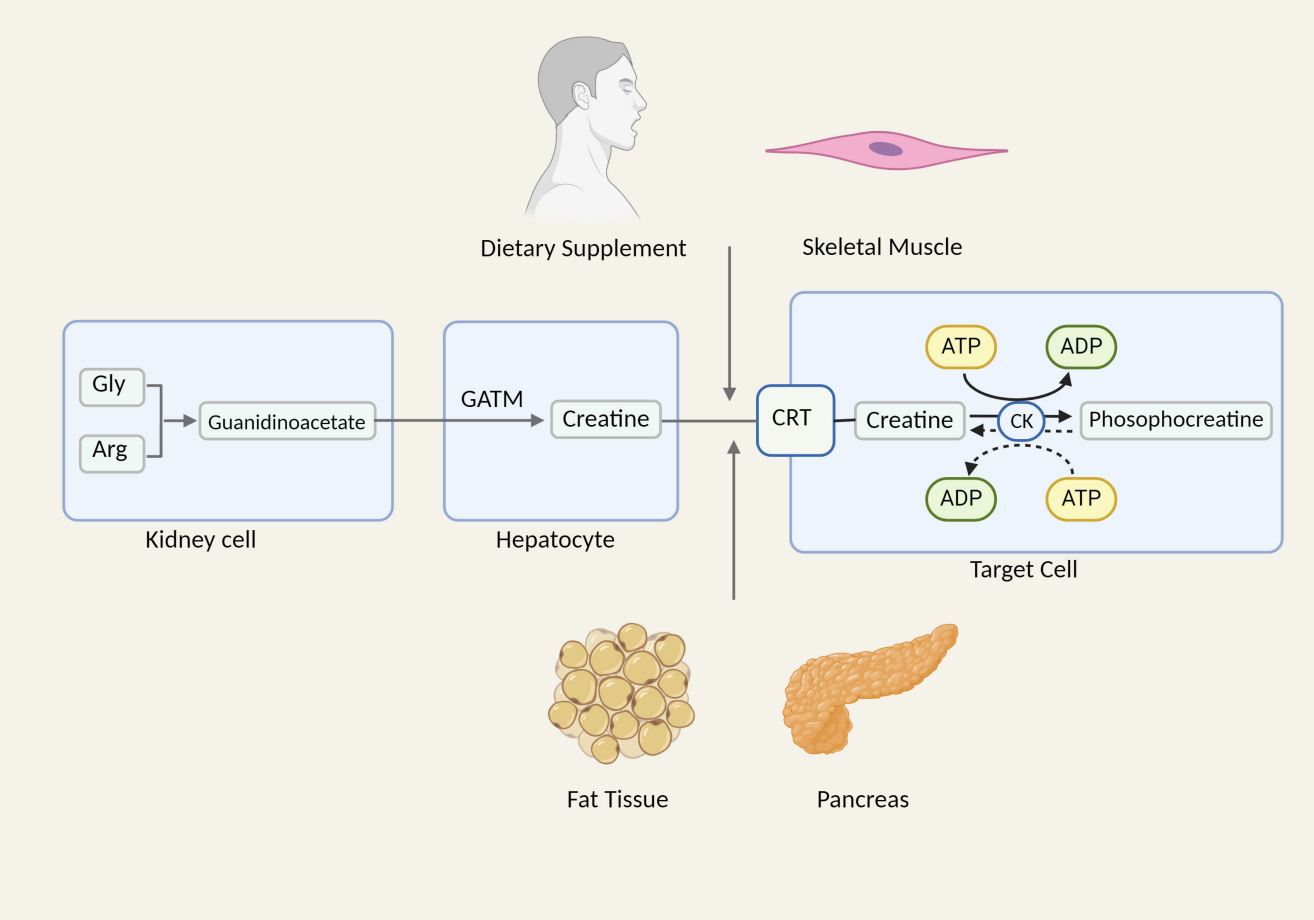
The synthesis and biological effects of creatine. Gly, glycine; Arg, arginine; CRT, creatine transporter.

Creatine kinase serves as the central regulating enzyme in cellular energy metabolism and is extensively present in muscle, brain, renal tubules, and other high-energy-demanding tissues and organs (78). Within the cell, creatine and creatine phosphate undergo reciprocal conversion through the control of creatine kinase, prompting the shuttle of high-energy phosphates at the site of ATP production, and providing support for mitochondrial and cellular energy turnover (79). Creatine, as a substrate for the reaction catalyzed by creatine kinase, is mostly present in muscle tissue and synthesized by the renal-liver axis. In the kidneys, arginine and glycine are synthesized into guanidine acetate, a precursor of creatine, under the action of glycinamide transferase (GATM) (80). After entering the peripheral circulation, guanidine acetate is absorbed by hepatocytes that produce guanidine acetate methyltransferase (GAMT), which then catalyzes the conversion of guanidine acetate to creatine. Following its export from the liver, creatine is taken up by cells that express creatine transporters and transformed by creatine kinases into creatine phosphate (81). Furthermore, creatine can be synthesized in other cell types, including fat, skeletal muscle, and pancreatic acinar cells, as well as absorbed by nutrition (82–84) (**Figure 2**).

### 4.1 Major depressive disorder

Studies have demonstrated a robust association between depression symptoms and fluctuations in levels of creatine and creatine kinase. Research findings indicated a correlation between the concentration of serum total creatine kinase and depressive symptoms in healthy women who are under stress (85). The volume of gray matter in the right medial superior frontal gyrus and the concentration of creatine in the prefrontal cortex were negatively correlated with the severity of depression, whereas the volume of gray matter in the right medial upper frontal cortex and the concentration of creatine in the medial prefrontal cortex were positively correlated (86), suggesting that increasing creatine concentration in the prefrontal cortex may improve the depressive symptoms of patients.

Numerous investigations on the amelioration of depression symptoms through creatine supplementation are already available, which amply supports the use of creatine in animal studies for the treatment of depression symptoms (87–92). One study showed that the administration of creatine helped improve depression-like behavior triggered by long-term corticosterone use (93). Systematic reviews have been conducted with an emphasis on creatine’s application in depression treatment (94). At present, many of the participants in the studies are women (95); In animal experiments, female rats exhibited a more favorable response to dietary creatine supplementation compared to male rats (96, 97), indicating that there might be a sex-dependent relationship between creatine and the treatment of depression symptoms. Studies of animal estrus cycle data revealed that the antidepressant effect of creatine on females mainly occurred in the proestrus and estrus, suggesting that ovarian hormones may have an impact on creatine’s therapeutic effects (96).

Previous review suggested that oligodendrocyte maturation and myelogenesis in the prefrontal cortex (PFC) were extremely responsive to stress experiences, and myelin-related transcriptional genes (MOG, MOBP, PLLP, PLP1, etc.) were significantly down-regulated when symptoms deteriorated. Exposing the mice to unpredictable chronic mild stress resulted in alterations in the transcription of approximately 30-40 genes, including genes linked with oligodendrocytes such as MBP, MOB, and CNP (98). Creatine can directly increase the production of mitochondrial ATP in the culture of oligodendrocyte line and protect caspase-dependent apoptosis of oligodendrocytes during myelin regeneration in the central nervous system (99), while the homozygous loss of GAMT, the rate-limiting enzyme of creatine production, leads to a significant reduction of mature oligodendrocytes in the corpus callosum and delays myelin formation (100). These findings suggested that creatine therapy may be related to the protection of nervous system oligodendrocytes and myelination.

### 4.2 Bipolar disorder

Current studies mostly have shown that patients with bipolar disorder exhibit notably elevated serum creatine kinase levels than healthy controls during the manic phase (101, 102). Hillbrand et al. investigated the relationship between creatine kinase and manic-like aggressive behavior and found that creatine kinase levels were strongly associated with aggressive behavior in patients treated with antipsychotics (103). This suggests that creatine kinase could serve as a valuable indicator for predicting violent behavior. Additionally, Meltzer et al. found that increased muscle-type creatine kinase levels precede aggression and may persist throughout the course of the disease (104).

In recent years, studies on the structural changes of brain tissue in patients with bipolar disorder have received extensive attention. Research has revealed that individuals with bipolar disorder have significantly reduced amounts of creatine kinase mRNA in hippocampal and prefrontal cortical regions compared to healthy individuals (105, 106). Du et al. used 31P magnetization transfer magnetic resonance spectroscopy to measure the forward response rate constant of creatine kinase. They discovered a noteworthy reduction in the forward response rate constant of creatine kinase in the bipolar disorder group when compared to the healthy control group. Furthermore, they pinpointed the precise molecular mechanism underlying bipolar disorder patients’ incapacity to refuel ATP in the brain during times of high energy demand (107).

A study involving phosphocreatine shuttling in the brain in bipolar disorder showed that in the frontal lobe and corpus callosum, high-energy phosphates are reduced in the form of phosphocreatine (108). Phosphocreatine levels in the frontal and occipital lobes, as well as the overall brain, are lower in adolescents and adults with bipolar disorder (109). This lends further credence to the hypothesis that bipolar disorder is associated with impaired mitochondrial activity or creatine metabolism. Nonetheless, certain research has demonstrated that there is no discernible variation in brain creatine levels between bipolar disorder patients and healthy individuals (110). The degree of physical and sexual abuse throughout childhood was linked to higher levels of creatine phosphate and lower creatine/creatine phosphate ratios in the anterior cingulate cortex in bipolar disorder patients (111).

Previous studies have showed that in addition to promoting and treating muscles, creatine supplementation may also promote brain health, such as cognitive processing, brain function, and repair after injury (112, 113). As a mitochondrial regulator, creatine monohydrate has been utilized as adjuvant therapy for individuals experiencing the depressive phase of bipolar disorder and has been reported to enhance cognitive function (114, 115). However, research has indicated that creatine can improve symptoms in patients with unipolar depression, while its use in bipolar depression may cause patients to transition to a manic phase (116). Bipolar disorder patients have been found to have lower levels of hippocampal myelination (117) and down-regulated brain oligodendrocytes as well as essential myelination-related genes (118) when compared to the control group.

## 5 Other biogenic amine metabolites and mood disorders

### 5.1 Taurine

Taurine, a sulfur-containing amino acid, is abundant in skeletal muscle (119–121) and plays a role in critical physiological processes including energy metabolism, lipid and carbohydrate metabolism and anti-inflammatory effects (122–124). As one of the essential amino acids for brain development, taurine has been found to be associated with the occurrence and development of depressive disorders. Previous studies showed that in depressed mice induced by chronic social failure stress, the level of taurine in extracellular fluid of the medial prefrontal cortex of the brain was significantly lessened. Additionally, the depression symptoms of the mice were significantly alleviated following taurine supplementation (125). In addition, taurine can lessen anxiety and enhance spatial memory in depressed rats, achieving the role of preventing depression-like behavior (126). The results suggested that the antidepressant effect of taurine may be related to the regulation of the hypothalamic-pituitary-adrenal (HPA) axis and the promotion of hippocampal neurogenesis, neuron survival and proliferation (126).

### 5.2 Betaine

Betaine, also known as trimethyl glycine, is mainly distributed in liver, kidney and brain (127). It can regulate human energy metabolism, relieve chronic inflammation (128), and help treat a range of illnesses, including obesity (129), diabetes (130, 131), Alzheimer’s disease (132) and other diseases. Furthermore, the research verified that betaine could ameliorate depression-like behavior in mice and has a neuroprotective effect on depressed mice generated by exposure to zinc oxide nanoparticles (133). Combining betaine with S-adenosine-methionine (SAMe) is more effective than SAMe alone in patients with mild to moderate depressive disorder (134, 135).

### 5.3 Choline

Choline is the hydrophilic head group of phosphatidylcholine and is involved in several physiological processes: the structural integrity of cell membranes, the transmission of lipid-derived signals and cholinergic neurotransmission and methylation (136). Choline supplementation might provide several beneficial outcomes, including diminishing the likelihood of cardiovascular events and breast cancer, as well as decreasing levels of inflammatory markers (137). In recent years, the connection between choline level and mood disorders has drawn a lot of attention. Dietary choline consumption was found to be inversely associated with the likelihood of experiencing depression symptoms in a cross-sectional study (138). Patients with refractory depressive disorder exhibited significantly elevated levels of choline in both the left and right hippocampus regions of the central nervous system, in comparison to first-episode patients and healthy individuals (139). Furthermore, a notable and favorable connection was discovered between the Hamilton Depression (HAMD) score and the choline/creatine (Cho/Cr) ratio in both the bilateral hippocampus areas and the left thalamus of patients with poststroke depression (140). After treatment, a significant decrease was detected in the Cho/Cr ratio of both the left thalamus and the bilateral hippocampal regions, raising the possibility that the aberrant choline metabolism in these regions is connected to post-stroke depression.

Manic individuals with greater average levels of choline in red blood cells are more likely to be unwell, have a worse prognosis, and a limited response to lithium treatment (141). Patients who do not respond to lithium demonstrate higher amounts of choline in the dorsolateral prefrontal lobe in comparison to patients who do respond positively to lithium (142). In addition, in contrast to controls, bipolar disorder patients had a considerably greater Cho/Cr-PCr ratio in the right cingulate cortex (143). The choline/creatine ratio in anterior cingulate cortex in adolescents with depressive disorder is significantly higher than that in patients with bipolar disorder (144), indicating that the total choline in anterior cingulate cortex may serve as a biomarker for the diagnosis of mood disorders in adolescents.

## 6 Associations between biogenic amine metabolites

The relationship between different biogenic amine metabolites remains uncertain. Taurine and amino acid metabolites such as creatine and histamine may work together through the same solute carrier superfamily. The SLC6 family, consisting of taurine and creatine transporters, is expressed in an array of tissues and is capable of actively transporting substrates on biofilms via gradients in sodium ion concentration (145). According to the study of Kurtz, J. A. et al., taurine supplementation can promote muscle regeneration and increase strength in male athletes (146), suggesting that exogenous taurine supplementation may affect creatine metabolism. Moreover, taurine and creatine have been found to present a time - and space-dependent imbalance in the brains of Alzheimer’s disease rats (147). Taurine supplementation throughout gestation can increase the N-acetyl aspartic acid (NAA)/creatine (Cr) ratio related to the number of neurons. It can also decrease the ratio of choline (Cho) to creatine (Cr), which is related to the number of glial cells. This improvement in hippocampal neuronal metabolism suggests that the balance between taurine and creatine may be linked to cognitive function (148). A previous study found that gut microbiota derived metabolites taurine, histamine, and spermine maintain the balance between host and gut microbiota by co-regulating NLRP6 inflammasome signaling, epithelial IL-18 secretion, and downstream antimicrobial peptide (AMP) profiles (149).

Choline, which is an organic penetrant and methyl donor in carbon metabolism (127), is converted to betaine, an oxidized byproduct. Betaine is a crucial neurotransmitter in a number of physiological functions, including memory retention and muscular control (150). A study in obese patients showed that choline and betaine supplements specifically raised histidine levels while simultaneously lowering taurine, acetic acid, and beta-hydroxybutyric acid levels, suggesting that choline and betaine supplements may have a beneficial effect on the citric acid cycle and can assist in the treatment of obesity-related diseases (151).

## 7 Other biological mechanisms of mood disorders interacting with biogenic amine metabolites

### 7.1 Inflammation

It has been discovered that inflammation is linked to stress-related mental diseases, such as major depressive disorder and bipolar disorder (152). Previous studies have shown that proinflammatory cytokines and acute phase proteins in blood were increased in depressive patients compared to healthy controls (153), and antidepressant treatment can considerably decrease the levels of IL-6, TNF, IL-10 and CCL2 in peripheral blood (154, 155). Furthermore, bipolar disorder patients also showed elevated serum levels of IL-2, IL-4, and IL-6 (156).

Inflammatory factors can act on the kynurenine pathway, thus affecting the occurrence of depression. Excessive activation of the kynurenine pathway results in disruptions in the levels of neurotoxic/neuroprotective compounds, particularly 3-HK/QA/Kyna. Studies have shown that patients treated with IFN-alpha have reduced serum tryptophan levels, increased Kyn levels and Kyn/Trp ratio activity, and increased depressive symptoms (157). Besides, antidepressants with anti-inflammatory properties suppress the activation of IDO by reducing levels of pro-inflammatory cytokines in immune-activated individuals, thereby aiding in the alleviation of depressive symptoms (158). The application of saffron extract can reduce depression-like behavior in mice through modulating the expression of KYN-related neurotoxicity (159).

Studies have shown that acute inflammation induced by lipopolysaccharides may lead to increased histamine and decreased serotonin in the central nervous system, leading to depression-like behaviors (160, 161). In addition, the utilization of creatine supplements has been shown to effectively alleviate many inflammation-related conditions, such as cancer (162) and osteoarthritis (163). Dietary creatine supplementation can delay the emergence of tumors in mice by regulating CD8+ T cell immunity (164). Zhang et al. demonstrated that betaine could facilitate the conversion of microglia from the M1 phenotype to the M2 phenotype by suppressing the activation of NLRP3 inflammasome, thus reducing the depressed-like behavior induced by lipopolysaccharide in mice (165).

### 7.2 Gut microbiota

A growing number of studies has demonstrated the link between mood disorders and alterations in the gut microbiome. It was widely acknowledged that proinflammatory bacteria were enriched and anti-inflammatory bacteria decreased in the gut of depressive patients, further strengthening the inflammatory hypothesis of depression (166). Metabolites derived from gut microbes are also strongly connected to mood disorders (167–170).

Increasing evidence suggests that kynurenine and its downstream metabolites, occurring in the gut, play an important role in regulating immune cell function, intestinal inflammation, and altering the production and inhibition of inflammatory cytokines (171, 172). Rats that received fecal microbiota transplants from depressive patients showed more pronounced depressive symptoms, associated with higher circulating kynurenine and kynurenine/tryptophan ratios (173). Probiotics, including L. helveticus R0052 and B. longum R0175, have been shown to have anxiety-reducing benefits in rats and positive psychological effects in healthy human volunteers (174).

Symbiotic microbes in the gut can produce histamine and related compounds under physiological conditions, suggesting that intestinal histamine might play a regulatory role in maintaining intestinal barrier function (149, 175, 176). Diets rich in polyunsaturated fatty acids, short-chain fatty acid and vitamin A have a protective effect against chronic stress in mice, while histamine deficiency can block this effect (177, 178).

The gut microbiome converts choline into several compounds, including trimethyl glycine (betaine) and trimethylamine (179). In addition, choline can undergo metabolism to produce hepatotoxic methylamines, such as dimethylamine, trimethylamine (TMA), and trimethylamine N-oxide (TMAO) (180). A high-fat diet alters the way choline is processed, leading to an increase in methylamine production (181). Furthermore, patients with major depressive disorder had higher levels of TMAO compared to healthy individuals, indicating that gut microorganisms can influence choline metabolism and are suspected of depressive symptoms in patients (182).

Multiple studies have shown dysregulation of intestinal taurine metabolism is significantly associated with anxiety and depression symptoms (183), and remedies using medication or modifications to the gut microbiota can ameliorate these symptoms by enhancing taurine metabolism (184, 185).

### 7.3 Brain-derived neurotrophic factor

Brain-derived neurotrophic factor (BDNF) binds to the tyrosine kinase receptor B (TrkB) receptor, which is directly involved in neurite growth, neuroplasticity and phenotypic maturation, and is crucial for the development and plasticity of synapses (186). The change of BDNF expression is closely related to normal and pathological aging (187), and has been verified to have a close correlation with mood disorders (188).

It is reported that the histamine H3R antagonist thioperamide hinders microglia activity and inflammatory response, and improves neurogenic injury and cognitive dysfunction by enhancing histamine release (189, 190). Besides, H3R antagonist s38093 was found to reverse the age-related effects of BDNF in the hippocampus of aging mice and increased hippocampal neurogenesis in adults, providing an innovative strategy to address cognitive deficits (191).

The mixture of creatine and taurine can trigger a notable rise in the expression of BDNF in stressed rats, and its antidepressant effect is related to the increase of behavior pattern mediated by extracellular regulated protein kinases (ERK)/BDNF pathway and mTORC1 signal pathway (192, 193). The presence of a specific genetic variation (BDNF rs6265) in the BDNF gene has a notable two-way interaction with creatine in bipolar disorder patients, suggesting that the pathogenesis of bipolar disorder may be related to the metabolism of the anterior cingulate cortex (194).

Taurine restores cognitive function and memory in rats exposed to toxic chemicals by stimulating N-methyl-D-aspartate (NMDA) receptors and elevating the expression of BDNF (195, 196), and counteracts the negative effects of cAMP-PKA-CREB signaling pathway on behavior and cognitive function of rats (197). Furthermore, taurine efficiently suppresses oxidative stress, neuroinflammation, and apoptosis in stressed rats by restoring BDNF expression and blocking the NF-κB signaling pathway (198).

### 7.4 Circadian rhythm

The disruption of rhythm is firmly connected with mood, sleep and physiological activities, and is one of the core symptoms of mood disorders, indicating that circadian rhythm disturbance is an important factor in the pathogenesis of mood disorders (199). The circadian system helps regulate a variety of vital functions, such as memory formation and maintenance, emotional regulation, cognitive function, and neuroplasticity (199, 200), via controlling the production of the CLOCK protein. Clock protein resides in various brain regions such as the hippocampus and amygdala (201), and can regulate the monoaminergic system in each region, thus regulating emotion-related behaviors (202, 203).

Tryptophan and kynurenine, as substrates for melatonin generation, could affect the synthesis of melatonin and the circadian rhythm, thereby impacting cognition and mood (204). In bipolar disorder, overactivation of the kynurenine pathway result in reduced tryptophan-produced melatonin, which leads to emotional symptoms and disruption of circadian rhythms (205). In addition, the kynurenine pathway and melatonin production may also be related to BDNF production, suggesting that the emergence of bipolar disorder may include a more intricate process.

Melatonin can interact with histamine signaling pathways and ultimately affect circadian rhythms (206). Previous studies have shown that histamine is associated with behavior and sleep-wake control (207), and is considered to be a downstream target of circadian action (208). Organic cation transporter 3 (OCT3) facilitates the transmission of histamine in mast cells and is linked to the regulation of the CLOCK gene, indicating that OCT3 plays a role in controlling the body’s internal clock (209). Changes in circadian rhythms can lead to modifications in the synthesis and transport of histamine, hence causing alterations in mood and behaviors.

## 8 Conclusion

Studies have shown that compared with healthy people, patients with mood disorders present notable variations in the biogenic amine metabolites in the peripheral and central nervous system. Future investigations should concentrate on the molecular pathway that links biogenic amine metabolites to mood disorders.

The two branches of kynurenine metabolism in the brain exhibit neuroprotective and neurotoxic effects, respectively, and the underlying mechanism of disease may be the equilibrium between the two branches, rather than the absolute levels of their metabolites. The disruption of circadian rhythm and melatonin synthesis may be attributed to the imbalance between the neurotoxic and neuroprotective pathways of kynurenine. Anti-inflammatory antidepressants may help to improve this imbalance.

Creatine, as a mitochondrial regulator, has been used to treat depressive disorders and bipolar depression, but there is a risk of transforming bipolar depression into bipolar mania. Previous studies have shown that patients with bipolar disorder and depressive disorder exhibit a decrease in the expression of genes associated with the development of oligodendrocytes and myelin. Additionally, creatine supplementation has been shown to protect oligodendrocytes and enhance myelin formation, raising the possibility that mood disorders may be caused by the creatine-related oligodendrocyte myelin pathway. The study on the mechanism of creatine as an adjunct therapy for depression can not only offer a fresh perspective on the treatment of depression, but also serve as a guide for managing depressive phase of bipolar disorder. In addition, peripheral creatine kinase levels also differed in patients with unipolar depression and bipolar disorder. Exploring the pathogenesis of peripheral creatine and creatine kinase pathway in mood disorders is helpful to identify new targets for the treatment of mood disorders.

Furthermore, biogenic amine metabolites are generated in peripheral tissues, such as liver and kidney, and it has been established that biogenic amine metabolites interact peripherally with the molecular processes of mood disorders, including gut microbiota and inflammation. In the future, studying the connections between peripheral signaling molecules and different brain regions—such as blood-brain barrier crossing and hormone axis changes—may be crucial to understanding the molecular foundations of mood disorders.
